# The interplay between hunting rate, hunting selectivity, and reproductive strategies shapes population dynamics of a large carnivore

**DOI:** 10.1111/eva.13253

**Published:** 2021-06-02

**Authors:** Joanie Van de Walle, Fanie Pelletier, Andreas Zedrosser, Jon E. Swenson, Stéphanie Jenouvrier, Richard Bischof

**Affiliations:** ^1^ Département de biologie & Centre for Northern Studies Université de Sherbrooke Sherbrooke QC Canada; ^2^ Biology Department Woods Hole Oceanographic Institution Woods Hole MA USA; ^3^ Department of Natural Sciences and Environmental Health University of South‐Eastern Norway Bø i Telemark Norway; ^4^ Institute of Wildlife Biology and Game Management University of Natural Resources and Life Sciences Vienna Austria; ^5^ Faculty of Environmental Sciences and Natural Resource Management Norwegian University of Life Sciences Ås Norway

**Keywords:** brown bear, harvest regulation, harvest‐induced selection, multistate population model, population dynamics, reproductive strategies

## Abstract

Harvest, through its intensity and regulation, often results in selection on female reproductive traits. Changes in female traits can have demographic consequences, as they are fundamental in shaping population dynamics. It is thus imperative to understand and quantify the demographic consequences of changes in female reproductive traits to better understand and anticipate population trajectories under different harvest intensities and regulations. Here, using a dynamic, frequency‐dependent, population model of the intensively hunted brown bear (*Ursus arctos*) population in Sweden, we quantify and compare population responses to changes in four reproductive traits susceptible to harvest‐induced selection: litter size, weaning age, age at first reproduction, and annual probability to reproduce. We did so for different hunting quotas and under four possible hunting regulations: (i) no individuals are protected, (ii) mothers but not dependent offspring are protected, (iii) mothers and dependent offspring of the year (cubs) are protected, and (iv) entire family groups are protected (i.e., mothers and dependent offspring of any age). We found that population growth rate declines sharply with increasing hunting quotas. Increases in litter size and the probability to reproduce have the greatest potential to affect population growth rate. Population growth rate increases the most when mothers are protected. Adding protection on offspring (of any age), however, reduces the availability of bears for hunting, which feeds back to increase hunting pressure on the nonprotected categories of individuals, leading to reduced population growth. Finally, we found that changes in reproductive traits can dampen population declines at very high hunting quotas, but only when protecting mothers. Our results illustrate that changes in female reproductive traits may have context‐dependent consequences for demography. Thus, to predict population consequences of harvest‐induced selection in wild populations, it is critical to integrate both hunting intensity and regulation, especially if hunting selectivity targets female reproductive strategies.

## 1 INTRODUCTION

Harvesting of wild animal populations serves economic, cultural, and management purposes, but when exerted at a high rate, it can threaten population persistence (Jackson et al., 2001) and induce trait changes in life history, morphology, and behavior (Palkovacs et al., 2018). Human harvest constitutes a unique form of “predation” that fundamentally differs from “natural predation,” because harvest mortality is often higher than natural mortality and not always directed toward individuals that are most vulnerable to natural mortality (Allendorf & Hard, 2009; Darimont et al., 2015; Festa‐Bianchet, 2003). Because of this, human harvest has emerged as an important driver of trait change in the wild (Darimont et al., 2009; Palumbi, 2001), inducing selective pressures that vary both in strength and in direction, depending on harvest levels and practices, as well as on the phenotypes being targeted (Darimont et al., 2015). Harvest‐induced selection on life‐history, morphological, and behavioral traits has been documented in both fishery and hunting systems (Allendorf & Hard, 2009; Boyce, 1981; Leclerc et al., 2017; Palkovacs et al., 2018; Van de Walle et al., 2018).

In addition to its direct effect on population growth rate, harvest can affect population structure and induce changes in phenotypic traits and behavior, which thus indirectly influence population growth rate (Gosselin et al., 2015; Milner et al., 2007; Pelletier et al., 2007). Harvest‐induced selection on traits linked to female reproductive performance is likely to have the greatest impact on the dynamics and persistence of populations. For instance, overfishing and harvest‐induced selection on body mass and size, strong drivers of individual performance in fish, are expected to lead to earlier sexual maturation at smaller sizes and reduction in population biomass (Jørgensen et al., 2007). Regardless of the mechanism generating them, phenotypic changes within fish populations have also been shown to have larger scale impacts (Fenberg & Roy, 2008). For example, the reduction in body size and egg production in Pacific salmon (*Oncorhynchus* spp.) over the past 60 years in Alaska resulted in reduced marine‐derived nutrient transport inland, with consequences for ecosystem functioning (Oke et al., 2020).

In long‐lived mammals, hunting‐induced selection commonly affects male secondary sexual traits, such as antlers, horns (Coltman et al., 2003; Jachmann et al., 1995; Pigeon et al., 2016), and body mass (Tenhumberg et al., 2004). However, hunting‐induced selection on these traits is likely to have limited consequences for population dynamics of such species due to the weak correlation between body mass (or correlated secondary sexual traits value), and reproductive performance in mammals (Kuparinen & Festa‐Bianchet, 2017), compared with fishes. Hunting‐induced selection acting directly on female reproductive traits has a much greater potential to affect population dynamics (Rughetti & Festa‐Bianchet, 2014; Servanty et al., 2011), although it has rarely been investigated (but see Proaktor et al., 2007; Rughetti & Festa‐Bianchet, 2014). Therefore, a step forward in our understanding of the large‐scale impacts of harvest would be facilitated by an evaluation of the demographic effects of changes in female reproductive traits in general, but especially in long‐lived species.

Female reproductive traits are often the target of harvest‐induced selection due to harvest intensity, regulations, and harvester preferences. Selection on female reproductive traits is generated by nonexclusive mechanisms that may act simultaneously. On the one hand, high rates of mortality should select for faster life histories and favor individuals that invest earlier and more into reproduction (Olsen et al., 2004; Stearns, 1992). This may explain why wild animal populations of the same species experiencing different levels of mortality often show contrasting life‐history strategies (Servanty et al., 2011; Zedrosser et al., 2011). Modeling and empirical studies have revealed that an increase in extrinsic mortality can select for earlier age at maturation, higher probability to reproduce, and increased litter/clutch size (Olsen et al., 2004; Proaktor et al., 2007). On the other hand, the nonrandom and systematic removal of specific phenotypes from a population due to hunting can also generate selection toward “shielding” traits (i.e., traits that afford a certain level of protection to individuals). Hunting regulations often aim at directing the harvest toward (or away from) individuals with specific traits within a population to achieve a management goal, for example, to manipulate the population growth rate. As such, hunting regulations can create harvest biases and, intentionally or not, induce selectivity (Bunnefeld et al., 2009; Festa‐Bianchet, 2003; Hengeveld & Festa‐Bianchet, 2011; Leclerc et al., 2016; Mysterud, 2011). In the case of reproductive traits, such hunting selectivity can affect the fitness pay‐off of different female reproductive tactics (Rughetti & Festa‐Bianchet, 2014; Van de Walle et al., 2018).

A common practice in the management of large mammal populations is to protect the female segment of the population to ensure population viability, because the survival and reproduction of prime‐aged females have the greatest potential to affect population growth, size, and fluctuations therein (Gaillard et al., 1998; Pelletier et al., 2011). In species where it is difficult to differentiate between females and males from a distance, protection of females is often achieved through the protection of family groups (Miller, 1990), as males generally do not provide parental care in mammals (Clutton‐Brock, 1991). In addition, the killing of mothers and dependent young may cause ethical concerns that often motivate the extension of the legal protection to dependent young. Family groups shield their members against hunting under such regulations, and selection on traits increasing the duration and frequency of the formation of family groups (e.g., age at first reproduction, reproductive rate, weaning age) can be expected. Even in the absence of regulations to protect offspring, hunters sometimes voluntarily refrain from killing members of a family group (Nilsen & Solberg, 2006; Rughetti & Festa‐Bianchet, 2011). Moreover, when hunting of dependent offspring is allowed, producing offspring has also been suggested to shield mothers against hunters, as hunters will shot offspring first (Ericsson, 2001). Despite the widespread application of protective measures for the reproductive segment of hunted populations, there is still little empirical and theoretical evidence of their consequences for population dynamics.

**FIGURE 1:**
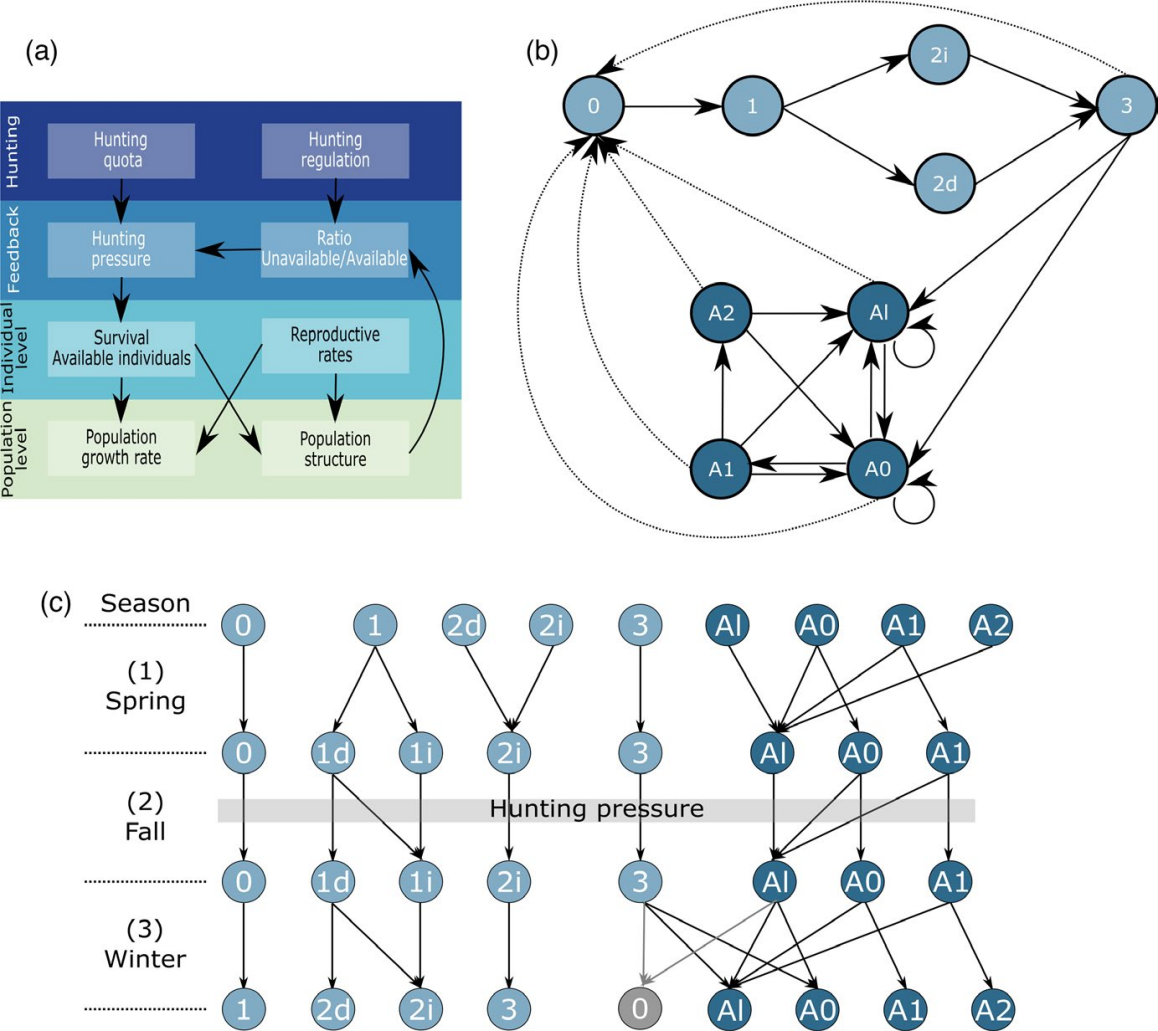
(a) Diagram showing the multi‐level processes implied in the population dynamics of the brown bear population in south‐central Sweden. At the hunting level, hunting quotas directly affect hunting pressure on bears. Hunting regulations, by dictating which categories of individuals can and cannot be hunted, affect the ratio between the number of bears unavailable and the number of bears available for hunting (feedback level). This ratio further affects hunting pressure on bears available for hunting. Hunting pressure on available bears affects their survival rate, which, combined with reproductive rates (individual level), affects both population growth rate and population structure (population level). The population is further regulated by a feedback loop (feedback level) as changes in population structure can affect the ratio of unavailable to available bears and feedback on hunting pressure on available bears. (b) Annual life cycle of the brown bear. Transitions between age and state stages occur over one year, from den emergence at time *t* until den emergence at time *t* +1. Transitions from a given stage to another are represented by solid arrows, and indirect contributions of a given stage to the cub stage through fertilities are represented by dotted arrows. Definitions: 0 = female cubs, 1 = female yearlings, 2*i* = independent two‐year‐old females, 2*d* = dependent two‐year‐old females, 3 = three‐year‐old females, *Al* =solitary adult females, *A*0 = adult females with cubs, *A*1 = adult females with dependent yearlings, *A*2 = adult females with dependent two‐year‐olds. (c) Periodic (seasonal) life cycle graph of the brown bear with three seasons: (1) spring (April–July; mating period), (2) fall (August–October; hunting period), and (3) winter (November–March; denning period). Individuals transit between the seasonal states conditional on survival, but also on reproductive rates through the probability to be weaned early or to wean early yearlings in the spring, the probability to give birth and emerge from the den the following spring with cubs as three‐year‐olds, and the probability to emerge from the den in the spring as adults with cubs. The indirect contribution of females to the cub (0) stage the following year is represented in gray. Yearlings (1) are all dependent (*d*) upon their mother at den emergence in the spring but can become independent (*i*) if they are weaned in the spring or if their mother dies in the following fall or winter. Dependent two‐year‐olds (2*d*) at den emergence are weaned in the spring and are thus independent (2*i*) in the fall along with other two‐year‐olds that were already weaned as yearlings in the previous year. Adult females (*A*) can become solitary (*Al*) the following season if they have weaned their cubs (in the spring only) or have lost their litter (all seasons) of cubs or yearlings. Hunting pressure is calculated as hunting mortality rate (*h*) based on the proportion of individuals unavailable for hunting at the beginning of the fall (hunting season), which is conditional on the state of individuals (dependent *vs* independent for subadults, and solitary *vs* with offspring for adults) at this time

Changes in environmental conditions or perturbations, such as hunting, can influence populations via feedback mechanisms (Lachish et al., 2020). Feedback loops in populations occur when demographic rates depend on current population properties (e.g., population size or composition). As the latter change in time, due to for example environmental changes, so does demographic rates and population dynamics (Kokko & López‐Sepulcre, 2007). Examples of feedbacks in demography include density dependence (Coulson et al., 2008; Rughetti & Festa‐Bianchet, 2014), frequency dependence (Jenouvrier et al., 2010), ecological feedbacks (Ransom et al., 2014), and eco‐evolutionary feedbacks (Govaert et al., 2019). A sustainable management practice is to set hunting quotas based on population censuses and as a proportion of the population that can be harvested annually (e.g., Andrén et al., 2020). This implies that changes in the phenotypic or genetic composition of the population (e.g., shift in reproductive trait values) or in population management (e.g., shift in target individuals) can affect the proportion of individuals legally protected from hunting and ultimately redirect and exacerbate the hunt toward the remaining, unprotected ones. In such systems, population composition can feedback on population dynamics through frequency‐dependent nonlinearity (Caswell, 2008) between the frequency of protected (unavailable) individuals and the survival rates of available individuals (Figure 1a).

Here, we took advantage of the long‐term and individual‐based monitoring of brown bears (*Ursus arctos*) in Scandinavia to identify the main processes by which hunting selectivity may impact population growth rate. To address this, we developed a state‐of‐the‐art multistate dynamic, frequency‐dependent matrix population model. Other main features of the model are cause‐specific mortality rates, intertwined fates between mother and offspring, and stage transitions dependent on reproductive traits. Using a demographic approach, we analyzed the model with five specific objectives. First, we characterized the brown bear population dynamics under observed conditions, accounting for frequency dependence. Second, we evaluated the impact of changes in hunting quotas on the population growth rate. Third, assuming potential selection for productivity and shielding traits, we predicted the consequences for population growth rate of changes in four reproductive traits: (1) offspring age at weaning (the probability to wean offspring early, that is, after 1.5 years of maternal care in brown bears), (2) age at first reproduction (the probability to mate at the age of three years and produce cubs at the age of four years), (3) reproductive rate of adult females (an adult female's probability to produce cubs), and (4) number of offspring produced (litter size). We expected a different contribution to population growth rate for each reproductive trait, with adult female reproductive rate having the greatest contribution, as is typically the case for large mammals (Gaillard et al., 1998). Fourth, we evaluated the impact of four hunting regulation scenarios along a gradient of legal protections afforded to members of family groups: (i) no individual is protected, (ii) mothers but not offspring are protected, (iii) mothers and offspring of the year (cubs) are protected, and (iv) entire family groups are protected (i.e., mothers and dependent offspring of any age) (e.g., Swenson et al., 2017). We predicted that an increase in hunting quotas should reduce population growth, but that this reduction would be mitigated by changes in female reproductive traits because such traits have the potential to “shield” females against harvest. Finally, we assessed the interactive effects of simultaneous changes in reproductive traits and hunting quotas under different hunting regulations. We expected that the mitigating effect of changes in reproductive rates on population growth would be exacerbated under higher levels of protection afforded to females as hunting pressure on the remaining available individuals would increase along with the benefits or being in a protected category of individuals.

## 2 MATERIALS AND METHODS

### 2.1 Study species, population, and system

The brown bear is a large, nonsocial carnivore widely distributed across Europe, North America, and Asia (Schwartz et al., 2003). Brown bears typically have a slow life‐history strategy (Steyaert et al., 2012); however, reproductive rates vary greatly among populations, contingent mainly on resource availability (Nawaz et al., 2008). Brown bears have been and still are hunted in several populations (Zedrosser et al., 2001), sometimes at very high intensities, such as in Scandinavia (Swenson et al., 2017).

Our study population of brown bears was located in Dalarna and Gävleborg counties in south‐central Sweden (~61^o^N, 14^o^E). The mean bear density in our study area was estimated at ~30 bears/1000 km^2^ in 2002 (Solberg et al., 2006). The brown bears’ mating season in our study area is in May–July, with a peak in the first week of June (Dahle & Swenson, 2003). During hibernation, which spans from the end of October until the end of April for adult females (Friebe et al., 2001), pregnant females give birth to 1–4 cubs (median = 2 cubs) in January and start lactation while in the den. Survival of cubs‐of‐the‐year (hereafter referred to as cubs) is relatively high, except in the spring, when the risk of sexually selected infanticide (SSI, the killing of unrelated offspring by males to gain access to reproduction with females; Hrdy, 1979) is high (Gosselin et al., 2017; Swenson et al., 1997). After litter loss due to SSI in the spring, victimized females soon resume estrus (Steyaert et al., 2014) and can give birth to a new litter the following year (Bellemain et al., 2006). In the absence of complete litter loss, females provide maternal care for at least 1.5 years, and yearling offspring can be weaned after den emergence in their second spring. Alternatively, females can continue maternal care for an additional year, for a total duration of 2.5 years, and two‐year‐olds are then weaned after den emergence in their third spring (Van de Walle et al., 2021). All offspring from the same family group separate simultaneously or within the same week (Dahle & Swenson, 2003). Whether or not a female weans her offspring as yearlings or two‐year‐olds depends on yearling mass in northern Sweden, but such a relationship has not been found in southern Sweden (Van de Walle et al., 2021).

Bear hunting is allowed throughout the species’ range in Sweden and anyone possessing hunting rights in an area and a weapon legal for big game hunting can shoot a bear (Bischof et al., 2008). Annual hunting quotas are set on a county basis and are determined by the national and county wildlife authorities (Swenson et al., 2017). Successful hunters are required by regulation to report their kill to the authorities and provide the location of the kill, body measurements, sex, and a tooth for age determination (Bischof et al., 2008). There is no limit to the number of bears an individual can shoot, as long as the county‐level quota has not been reached. Because there is little incentive for hunters to pass on an opportunity to kill a bear, bear hunting in Sweden is mostly considered as nonselective with regard to age, sex, and size (Bischof et al., 2009), although recent estimates show that hunting may now be slightly biased toward older males and larger individuals (Bischof et al., 2018; Leclerc et al., 2016). However, since 1986, all members of a family group of bears, that is, a female accompanied by dependent offspring of any age, have been afforded legal protection from hunting (Swenson et al., 2017). By providing a survival advantage to members of family groups, this regulation artificially selects for longer periods of mother–offspring associations (Van de Walle et al., 2018), and any other trait allowing individuals to form and remain in a family group is also expected to be under selection.

### 2.2 Bear monitoring

The brown bear population in southern Sweden has been monitored using radio‐telemetry since 1985. The objective of the monitoring program is to follow female bears, ideally from birth until death (Swenson et al., 1998). Bears were captured by darting (Dan‐Inject, Børkop, Denmark) with an immobilizing drug in the spring, soon after den emergence (Arnemo et al., 2011). Most bears were first captured as yearlings with their mother during the annual spring capture season. For ethical reasons, cubs were not captured. For bears of unknown age when captured, age was determined by analyzing annuli cementum widths of an extracted vestigial premolar (Matson et al., 1993). Captured individuals were measured (e.g., weight, head circumference), identified with a uniquely coded tattoo on the lower lip and a microchip transponder, and their sex was documented. All females were equipped with a VHF transmitter (Telonics, model IMP/400/L HC) implanted in the peritoneal cavity. From 2003 onward, female bears were also equipped with a GPS collar (GPS Plus; Vectronic Aerospace, Germany), except yearling females due to their rapid growth. After release, radio‐marked females were located from the ground or a helicopter a minimum of three times during their active period to assess their reproductive status (solitary, with cubs, or with yearlings) and the number of offspring was counted. Handling of study animals in the monitoring program was approved by the appropriate authorities and ethical committees: Swedish Board of Agriculture (no. 35‐846/03, 31‐7885/07, 31 11102/12), Uppsala Ethical Committee on Animal Experiments (no. C40/3, C47/9, C7/12), and Swedish Environmental Protection Agency (no. 412–7327–09 Nv). The monitoring program provided information on female reproductive traits (i.e., litter size, age at first reproduction, probability of adult females to produce, and offspring age at weaning). In Sweden, all bears killed legally (e.g., legal hunting, management kills, defense of life and property) must be reported to the management authorities. Death due to other reasons (e.g., natural deaths, vehicle and train collisions, illegal hunting) has also to be reported, although an unknown proportion of mortalities remains undetected (Bischof et al., 2008, 2009).

### 2.3 Population dynamics modeling

#### 2.3.1 Model including frequency‐dependent hunting mortality

We built a nonlinear matrix population model structured by age and state stages. The model projects the number of individuals within each stage, **n**, from year *t* to *t* + *1* based on the projection matrix **A** and the vector of parameters **θ**. The vector of parameters **θ** is function of the current population vector **n** (Caswell, 2001; Jenouvrier et al., 2010) because of the dependency of hunting mortality on the frequency (or proportion) of individuals unavailable for hunting in the population. Therefore, the population is projected as follows:

(1) $$n_{t+1}=A\left[\theta \left(n_{t}\right)\right]n_{t}$$

If we define hunting quota (q) as the proportion of the total population size ($N_{T,t}$) to be harvested each year, the annual probability that an available (unprotected) individual will die from hunting ($h_{t}$) is:

(2) $$h_{t}=q\times \left(1+\frac{N_{u,t}}{N_{a,t}}\right)$$

where $N_{T,t}$ is the sum of available ($N_{a,t}$) and unavailable ($N_{u,t})$ individuals at time *t*. It follows that, with a growing proportion of the population protected from hunting, hunting pressure (and thus the risk of being killed) increases for the remaining unprotected individuals. Availability to hunting depends on the state of an individual at the beginning of the hunting season (fall), which can differ from the one at the beginning of the spring. For example, a dependent yearling weaned in the spring becomes available for hunting as an independent yearling in the fall. To account for these seasonal transitions in our estimation of $N_{u,t}$ and $N_{a,t}$, we built a periodic model with three seasons.

#### 2.3.2 Periodic model

We start by presenting the annual life cycle graph and then its season decomposition. Annual transitions are estimated assuming postbreeding censuses, that is, between den emergence at year *t* and den emergence at year *t*+1 to match with the sampling protocol of the monitoring program. The annual life cycle is based on females assuming a sex ratio of 1:1 within litters and includes 9 stages (Figure 1b): 5 juvenile and 4 adult stages, based on the age/state in which females are at den emergence at year *t*:

1. Cubs (0): female cubs born in January of a given year that are dependent on their mother during the entire year.
2. Dependent yearlings (1): after their second hibernation with their mother, female yearlings emerging from the den are still dependent upon their mother.
3. Dependent two‐year‐olds (2*d*): 2‐year‐old females that have hibernated with their mother for a third winter and are still dependent on their mother in early spring of their third year.
4. Independent two‐year‐olds (2*i*): 2‐year‐old females that have been weaned the previous spring.
5. Three‐year‐olds (3): independent 3‐year‐olds.
6. Lone adults (*A*l): females aged ≥ 4 years that are solitary.
7. Adults with cubs (*A*0): females aged ≥ 4 years that have mated the previous spring and have emerged from the den with cubs.
8. Adults with dependent yearlings (*A*1): females aged ≥ 5 years that hibernated for a second winter with their offspring.
9. Adults with dependent two‐year‐olds (*A*2): females aged ≥ 6 years that have not weaned their yearling offspring during the previous spring and have hibernated for a third winter with their offspring.

The life cycle graph is then further decomposed into seasonal transitions (Figure 1c). Seasons were defined based on key events in the annual life cycle of brown bears: spring (May 1–July 31) corresponds to the mating season, fall (August 1–October 31) to the period of hyperphagia prior to hibernation, and winter (November 1–April 30) to the hibernation season (Bischof et al., 2018). The population includes different stages at each season (Figure 1c) as it may happen with periodic models (Jenouvrier et al., 2010). The population matrix **M** *_i_* projects the population from one season to the other. The annual dynamics are given by the multiplication of the seasonal matrices:

(3) $$n_{t+1}=M_{3}M_{2}M_{1}n_{t}$$

Matrix **M**_1_ projects the population from the 9 stages (*i*) at the beginning of the spring (*s*) to the 8 stages at the beginning of the fall (Figure 1c), conditional on early weaning probability (a), probability to lose a litter of cubs (l), and stage‐specific survival ($S_{i}$) rates:

$$M_{1}=\left(\begin{array}{ccccccccc} S_{A0,s}\times S_{0,s} & 0 & 0 & 0 & 0 & 0 & 0 & 0 & 0\\ 0 & \left(1‐a\right)\times S_{A1,s}\times S_{1,s} & 0 & 0 & 0 & 0 & 0 & 0 & 0\\ 0 & a\times S_{1i,s} & 0 & 0 & 0 & 0 & 0 & 0 & 0\\ 0 & 0 & S_{2,s} & S_{2,s} & 0 & 0 & 0 & 0 & 0\\ 0 & 0 & 0 & 0 & S_{3,s} & 0 & 0 & 0 & 0\\ 0 & 0 & 0 & 0 & 0 & S_{Al,s} & l_{s}\times S_{Al,s} & a\times S_{Al,s} & S_{Al,s}\\ 0 & 0 & 0 & 0 & 0 & 0 & \left(1‐l_{s}\right)\times S_{Al,s} & 0 & 0\\ 0 & 0 & 0 & 0 & 0 & 0 & 0 & \left(1‐a\right)\times S_{Al,s} & 0 \end{array}\right)$$

Note here that survival of cubs is assumed conditional on their own survival and that of their mother. If weaned or if the mother died, yearlings become independent in the fall. Adult females can lose their litter due to weaning or the death of all offspring in the litter. All *A*2 females will wean their two‐year‐olds in the spring. All adult females that have lost their litter become solitary in the fall.

Matrix **M**_2_ projects the population from the 8 stages at the beginning of the fall (*f*) to the 8 stages at the beginning of the winter (Figure 1c), conditional on probability to lose a litter of cubs (l) or yearlings (y) and stage‐specific survival ($S_{i}$) rates:

$$M_{2}\left[n\right]=\left(\begin{array}{cccccccc} S_{0,f}\times S_{A0,f} & 0 & 0 & 0 & 0 & 0 & 0 & 0\\ 0 & S_{A1,f}\times S_{1d,f} & 0 & 0 & 0 & 0 & 0 & 0\\ 0 & \left(1‐S_{A1,f}\right)\times S_{1i,f} & S_{1i,f} & 0 & 0 & 0 & 0 & 0\\ 0 & 0 & 0 & S_{2,f} & 0 & 0 & 0 & 0\\ 0 & 0 & 0 & 0 & S_{3,f} & 0 & 0 & 0\\ 0 & 0 & 0 & 0 & 0 & S_{Al,f} & l_{f}\times S_{Al,f} & y_{f}\times S_{Al,f}\\ 0 & 0 & 0 & 0 & 0 & 0 & \left(1‐l_{f}\right)\times S_{A0,f} & 0\\ 0 & 0 & 0 & 0 & 0 & 0 & 0 & \left(1‐l_{f}\right)\times S_{A1,f} \end{array}\right)$$

In the fall, yearlings that have lost their mother become independent and adult females that have lost their litter become solitary; all other females remain in their previous stage conditional on survival. Except for cubs, survival rates in the fall of all other stages at time *t* include both mortality rates from hunting ($h_{t}$) and stage‐specific mortality rates from other causes ($w_{i,f}$) as additive causes of mortalities; $S_{i,f,t}=(1‐w_{i,f}‐h_{t}$). In **M**_2_, $h_{t}$ = $h_{t}$[**n**] and thus depends on population structure, following Equation 2 above. $N_{u,t}$ and $N_{a,t}$ are calculated as the number of females within the stages that are protected (i.e., “unavailable”) and not protected (i.e., “available”) from hunting at the beginning of the fall. Hunting regulations determine the stages which are afforded protection. For instance, in the case of protection of family groups, stages 0, 1*d*, *A*0, and *A*1 in the fall are unavailable, whereas stages 1*i*, 2, 3, and *Al* are available. Because the model is females‐based, it assumes that an increase in the frequency of unavailable females will spread hunting mortalities over the remaining available females only. Because the hunting quota is fixed based on total population size, and hunters cannot distinguish between males and females at a distance, hunting mortalities should be spread over males as well. Therefore, we included males in our calculations of $N_{u,t}$ and $N_{a,t}$ based on the female population vector, assuming a 1:1 sex ratio in the population. Availability of males was determined from their presumed stage as subadults, but since males do not contribute to parental care in brown bears, all adult males are available for hunting.

Matrix **M**_3_ projects the population from the 8 stages at the beginning of the winter (*w*) to the 9 stages at the beginning of the spring at year *t*+*1*, conditional on the probability of losing a litter of cubs (l) or yearlings (y), state‐specific fecundity ($f_{i}$), and survival ($S_{i}$) rates:

$$M_{3}=\left(\begin{array}{cccccccc} 0 & 0 & 0 & 0 & f_{3} & f_{\mathrm{Al}} & 0 & 0\\ S_{A0,w}\times S_{0,w} & 0 & 0 & 0 & 0 & 0 & 0 & 0\\ 0 & S_{A1,w}\times S_{1d,w} & 0 & 0 & 0 & 0 & 0 & 0\\ 0 & \left(1‐S_{A1,w}\right)\times S_{1i,w} & S_{1i,w} & 0 & 0 & 0 & 0 & 0\\ 0 & 0 & 0 & S_{2,w} & 0 & 0 & 0 & 0\\ 0 & 0 & 0 & 0 & S_{3,w}\times \left(1‐r_{3}\right) & S_{Al,w}\times \left(1‐r_{\mathrm{Al}}\right) & l_{w}\times S_{Al,w} & y_{w}\times S_{Al,w}\\ 0 & 0 & 0 & 0 & S_{3,w}\times r_{3} & S_{Al,w}\times r_{\mathrm{Al}} & 0 & 0\\ 0 & 0 & 0 & 0 & 0 & 0 & \left(1‐l_{w}\right)\times S_{A0,w} & 0\\ 0 & 0 & 0 & 0 & 0 & 0 & 0 & \left(1‐y_{w}\right)\times S_{A1,w} \end{array}\right)$$

Here again, yearlings that have lost their mother become independent and adult females that have lost their litter become solitary, all other females remain in their previous stage conditional on their survival. Females aged three years and solitary adult females will emerge from their den with cubs with a probability $r_{3}$ and $r_{\mathrm{Al}}$ and can contribute to the cubs stage the following spring through $f_{3}$ and $f_{\mathrm{Al}}$ (See Appendix S1: Tables S1 and S2, for detailed mathematical descriptions of the transitions).

#### 2.3.3 Model parameterization

**FIGURE 2:**
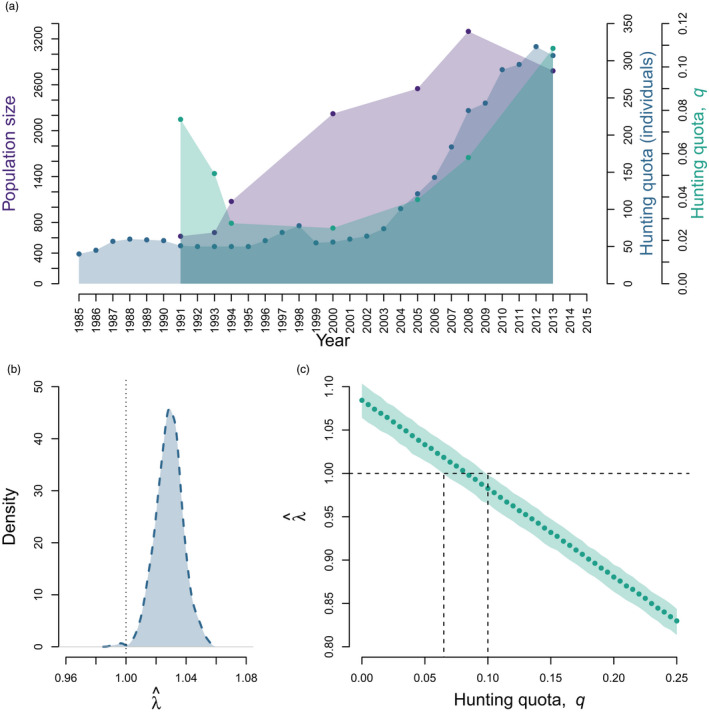
(a) Temporal changes in Swedish brown bear population size, hunting quotas (number of individuals allowed to be hunted annually), and hunting quotas, q (proportion of total population allowed to be hunted annually). Data were taken from Swenson et al. (2017) for the period 1985–2015, except for q, which was calculated here as hunting quotas (numbers) divided by population size during years when estimates of population sizes were available. The parameter q was calculated only for the years when both population size and hunting quotas (individuals) were available. (b) Population growth rate at equilibrium ($\hat{\lambda }$) based on observed vital rates, assuming q = 5.5%. (c) Changes in $\hat{\lambda }$ under simulated changes in hunting quota, q. Note that here a q value of 0.05 corresponds to a 5% quota. Bootstrapped distributions in (b) and (c) were obtained by randomly drawing parameters within their distribution of estimation 1000 times and under the current hunting regulation of protecting family groups. Only the 95% confidence interval is shown in (c). The dotted lines perpendicular to the x‐axis in (b) and the y‐axis in (c) indicate the threshold between population decline ($\hat{\lambda }$ < 1) and population growth ($\hat{\lambda }$ > 1). In (c), the dotted lines perpendicular to the y‐axis indicate the minimum and maximum hunting quotas leading to stable population ($\hat{\lambda }$ = 1)

We used 29 parameters (see Table 1 for a complete list with their source) to derive the demographic rates included in the transitions in our population matrices. With few exceptions, we parameterized our model with estimates from previously published studies on the Scandinavian brown bear population, covering the period 1985–2015 (Bischof et al., 2018; Swenson et al., 2017). These include season and cause‐specific survival rates and reproductive transitions presented recently by Bischof et al. (2018) as posterior samples with associated means and 95% credible intervals from a Bayesian multistate capture–mark–recapture model fitted to data from the same study population. The posterior samples for those parameters are available as part of the supplementary information to Bischof et al. (2018). Hunting quota, q, was estimated as the average annual hunting quota based on data from Swenson et al. (2017) between the period 1985–2013 (Figure 2a). Previous CMR parameter estimation did not account for the potentially different mortality rates between dependent and independent yearlings, which were suggested in Van de Walle et al. (2018). We thus empirically estimated mortality rates from hunting and other causes of yearlings, based on their dependency status during the yearling and two‐year‐old stages. Using data from 1990 to 2015, we estimated mortality rate in each season as the proportion of yearling deaths from each cause divided by the number of yearlings at the beginning of the season, assuming a detection probability of 1.

**TABLE 1:** Definitions, average estimates (with 95% posterior distributions), and elasticity values of the lower‐level parameters included in the frequency‐dependent dynamic population model of the Swedish brown bear population, 1985–2015. Because hunting mortality rate (*h*) of available categories of females (stages 1*i*, 2, 3, and *Al* under the protection of family groups) is frequency‐dependent and derived from the parameter q, we here only present information on q. Hunting mortality rate (*h*) of unavailable categories of females (stages 1*d*, *A*0, and *A*1) was set to 0 under the hunting regulation protecting members of family groups

Parameter	Description	Estimate [95% CI]	Back‐transformed estimate	Note	Reference	Elasticity
$S_{0,s}$	Survival probability of cubs during the spring	0.38 [0.10, 0.70]	0.594	For cubs with mothers of age 6–10 years old	Bischof et al. (2018)	0.070
$S_{0,f}$	Survival probability of cubs during the fall	5.38 [4.10, 7.03]	0.995	For cubs with mothers of age 6–10 years old	Bischof et al. (2018)	0.085
$S_{0.w}$	Survival probability of cubs during the winter	3.40 [2.81, 4.04]	0.968	For cubs with mothers of age 6–10 years old	Bischof et al. (2018)	0.086
$w_{1d,s}$	Mortality rate due to causes other than hunting for dependent yearling females during the spring	0.12 [0.03, 0.24]	–	–	Estimated in the present study	−0.004
$w_{1d,f}$	Mortality rate due to causes other than hunting for dependent yearling females during the fall	0.00 [0.00, 0.00]	–	–	Estimated in the present study	0.000
$w_{1d,w}$	Mortality rate due to causes other than hunting for dependent yearling females during the winter	0.00 [0.00, 0.00]	–	–	Estimated in the present study	0.000
$w_{1i,s}$	Mortality rate due to causes other than hunting for independent yearling females during the spring	0.09 [0.04, 0.14]	–	–	Estimated in the present study	−0.006
$w_{1i,f}$	Mortality rate due to causes other than hunting for independent yearling females during the fall	0.03 [0.00, 0.07]	–	–	Estimated in the present study	−0.002
$w_{1i,w}$	Mortality rate due to causes other than hunting for independent yearling females during the winter	0.01 [0.00, 0.03]	–	–	Estimated in the present study	−0.001
$w_{2,s}$	Mortality rate due to causes other than hunting for 2‐year‐old females during the spring	−4.49 [−6.47, −3.05]	0.011	–	Bischof et al. (2018)	−0.001
$w_{2,f}$	Mortality rate due to causes other than hunting for 2‐year‐old females during the fall	−5.03 [−5.88, −4.32]	0.006	–	Bischof et al. (2018)	−0.001
$w_{2,w}$	Mortality rate due to causes other than hunting for 2‐year‐old females during the winter	−4.02 [−4.57, −3.56]	0.018	–	Bischof et al. (2018)	−0.002
$w_{3,s}$	Mortality rate due to causes other than hunting for 3‐year‐old females during the spring	−3.99 [−5.65, −2.77]	0.018	–	Bischof et al. (2018)	−0.002
$w_{3,f}$	Mortality rate due to causes other than hunting for 3‐year‐old females during the fall	−5.03 [−5.88, −4.32]	0.006	–	Bischof et al. (2018)	−0.001
$w_{3,w}$	Mortality rate due to causes other than hunting for 3‐year‐old females during the winter	−4.02 [−4.57, −3.56]	0.018	–	Bischof et al. (2018)	−0.001
$w_{Al,s}$	Mortality rate due to causes other than hunting for solitary females during the spring	−3.91 [−4.74, −3.27]	0.020	For females of ages 6–10 years old.	Bischof et al. (2018)	−0.009
$w_{Al,f}$	Mortality rate due to causes other than hunting for solitary females during the fall	−5.03 [−5.88, −4.32]	0.006	For females of ages 6–10 years old	Bischof et al. (2018)	−0.003
$w_{Al,w}$	Mortality rate due to causes other than hunting for solitary females during the winter	−4.02 [−4.57, −3.56]	0.018	For females of ages 6–10 years old	Bischof et al. (2018)	−0.006
$w_{A0,s}$	Mortality rate due to causes other than hunting for females with cubs during the spring	−3.91 [−4.74, −3.27]	0.020	For females of ages 6–10 years old	Bischof et al. (2018)	−0.005
$w_{A0,f}$	Mortality rate due to causes other than hunting for females with cubs during the fall	−5.03 [−5.88, −4.32]	0.006	For females of ages 6–10 years old	Bischof et al. (2018)	−0.002
$w_{A0,w}$	Mortality rate due to causes other than hunting for females with cubs during the winter	−4.02 [−4.57, −3.56]	0.018	For females of ages 6–10 years old	Bischof et al. (2018)	−0.005
$w_{A1,s}$	Mortality rate due to causes other than hunting for adult females with yearling females during the spring	−3.91 [−4.74, −3.27]	0.020	For females of ages 6–10 years old	Bischof et al. (2018)	−0.002
$w_{A1,f}$	Mortality rate due to causes other than hunting for adult females with yearling females during the fall	−5.03 [−5.88, −4.32]	0.006	For females of ages 6–10 years old	Bischof et al. (2018)	0.000
$w_{A1,w}$	Mortality rate due to causes other than hunting for adult females with yearling females during the winter	−4.02 [−4.57, −3.56]	0.018	For females of ages 6–10 years old	Bischof et al. (2018)	−0.001
$r_{3}$	Probability that a 3‐year‐old female emerges from her den with cubs the next spring	−1.79 [−2.37, −1.24]	0.143	–	Bischof et al. (2018)	0.003
$r_{\mathrm{Al}}$	Probability that a solitary female emerges from her den with cubs the next spring	0.20 [−0.10, 0.48]	0.550	For females aged 6–10 years old	Bischof et al. (2018)	0.055
a	Probability that a female weans her litter of yearlings	0.65 [0.18, 1.13]	0.657	Joint probability of weaning and losing the entire litter of yearlings in the spring	Bischof et al. (2018)	0.012
$n_{{\text{cub}}_{s}}$	Number of cubs within a litter in the spring	2.37 [2.22, 2.53]	–	–	Bischof et al. (2018)	0.074
q	Hunting quotas	0.055		Proportion of total population hunted every year	Swenson et al. (2017)	−0.054

Early weaning probability (a) is the probability that a female accompanied by yearlings weans (or separates from) her cubs at this stage. A high value of a means reduced weaning age and interbirth interval and thus increased productivity. Probability to mate as a three‐year‐old ($r_{3}$) is the probability that a three‐year‐old female at time *t* mates in the spring and emerges from her den with cubs at four years old at time *t* + *1*. A high value of $r_{3}$ means earlier age at first reproduction and thus increased productivity. The probability to produce cubs for adult females ($r_{\mathrm{Al}}$) is the probability that an adult solitary female in the winter emerges from her den with cubs at time *t* + *1*, conditional on having had the opportunity to mate in the previous spring. A high value of $r_{\mathrm{Al}}$ means higher reproductive rates and productivity. Litter size ($n_{\text{cub}}$) is the total number of cubs produced, regardless of their sex, and thus ranges from 1 to 4. Assuming a 1:1 sex ratio at birth, half of this number will enter the female cub stage in our female‐based model. High $n_{\text{cub}}$ values also indicate high productivity.

#### 2.3.4 Model analyses and projections

##### Population dynamics under observed conditions

We parameterized the frequency‐dependent population model with observed parameters (Table 1) to estimate population growth rate under the current hunting regulation protecting members of family groups. In years when population size was estimated, hunting quotas (q, in %) ranged from 3 to 11%, with an average of 5.5% (Figure 2a). The parameter q was thus set at 5.5% in our simulations. Frequency‐dependent models eventually converge to an equilibrium population structure ($\hat{p}$) and population growth rate ($\hat{\lambda }$; Caswell, 2001). We thus inspected the temporal dynamics of 10 simulations using random initial population vectors over 100 years to identify time at convergence. Once convergence was reached, we derived $\hat{\lambda }$ as the dominant eigenvalue of our projection matrix $\hat{A[}\hat{p]}$. To account for uncertainty in parameter estimates, we randomly resampled each parameter estimate 1000 times from a normal distribution based on the 95% credible interval of the Bayesian posterior distribution of parameter estimates. This resulted in a posterior sample of 1000 $\hat{\lambda }$. Then, we assessed how the parameters influence population growth rate using perturbation analysis, adapted for nonlinear models (Caswell, 2008). To account for nonlinearity in our model, we conducted elasticity (i.e., the proportional effect on population growth rate of a proportional change in a parameter) analyses at equilibrium (Caswell, 2008; Jenouvrier et al., 2010 equations 16–17).

##### Impact of hunting quota

We evaluated the effect of hunting quotas on population growth by re‐estimating $\hat{\lambda }$ over simulated hunting quotas ranging from 0 to 25% by increments of 0.5%. Simulations were performed assuming legal protection of family groups. For each simulated hunting quota, we bootstrapped the procedure by randomly drawing the remaining parameters 1000 times within the parameter normal distribution to obtain a 95% confidence interval (CI) around the mean prediction.

##### Effects of changes in reproductive rates

We evaluated the effect of changes in four reproductive traits, that is, a (early weaning probability), $r_{3}$ (probability to mate at the age of three years), $r_{\mathrm{Al}}$ (probability to produce cubs), and $n_{\text{cub}}$ (litter size), on$\hat{\lambda }$. In our models, we allowed one reproductive parameter to vary at a time, while keeping the other parameters constant. We simulated probabilities (a, $r_{3}$, $r_{\mathrm{Al}}$) over the range 0–1 by increments of 0.01, whereas counts ($n_{\text{cub}}$) were simulated between 1 and 4 by increments of 0.01. Simulations were performed with q = 5.5% and assuming legal protection of family groups. As above, we bootstrapped the procedure by randomly drawing the remaining parameters 1000 times within the parameter normal distribution to obtain a 95% confidence interval around the mean prediction.

##### Effects of hunting regulation

We measured the impact of hunting regulation on population dynamics by recalculating $\hat{p}$ and $\hat{\lambda }$ under four hunting regulation scenarios: (i) no individual is protected, (ii) mothers but not offspring are protected, (iii) mothers and cubs are protected, and (iv) entire family groups are protected (i.e., mothers and dependent offspring of any age). Depending on the scenario considered, availability to hunting in the fall was redefined based on which category of individuals was afforded legal protection. The resulting hunting probability, *h*, was applied to the available categories of individuals only (Appendix S1: Table S3). To highlight contrasts in hunting regulations, we simulated two hunting quotas: (1) 5%, that is, a hunting quota close to the average observed in our study population, and (2) 20%, that is, an extreme hunting quota.

##### Interplay between quotas, reproduction, and regulation

Finally, we investigated the interactive effects of hunting quotas and reproductive traits, which would indicate potential for hunting‐dependent selective gradients on reproductive traits. We started by estimating the elasticities of reproductive parameters over hunting quotas ranging from 0 to 25% to identify linear relationships that would suggest that the potential for reproductive traits to influence population growth rate is hunting‐dependent. Then, we estimated $\hat{\lambda }$ for all combinations of hunting quotas and reproductive traits under the four scenarios of hunting regulations. Under each scenario, we varied one reproductive trait and one hunting quota at a time and applied the resulting *h* only to the stages available for hunting.

## 3 RESULTS

### 3.1 Population dynamics under observed conditions

Over the study period 1985–2015, using mean parameter values, the predicted $\hat{\lambda }$ was 1.029 (95% CI = [1.011, 1.045]). Of the 1,000 bootstrap iterations, only 0.3% of the simulations showed a population decline ($\hat{\lambda }$ < 1; Figure 2b). The parameters to which $\hat{\lambda }$ showed the greatest elasticity values were survival rates of cubs at each season (elasticity for *S_0_*_,_ *_s_*: 0.070; *S_0_*_,_ *_f_*: 0.085; *S_0_*_,_ *_w_*: 0.086), litter size (elasticity for $n_{\text{cub}}$: 0.074), probability for adult females to produce cubs (elasticity for $r_{\mathrm{Al}}$: 0.055), and hunting quota (elasticity for q: −0.054; Table 1).

### 3.2 Impact of hunting quota

$\hat{\lambda }$ declined sharply and linearly with increasing hunting quotas (Figure 2c). Within the hunting quotas interval [0.065, 0.100], the population was stable ($\hat{\lambda }$ = 1.0). Hunting quotas below this and above this range would result in population growth or decline, respectively.

### 3.3 Effects of changes in reproductive rates

**FIGURE 3:**
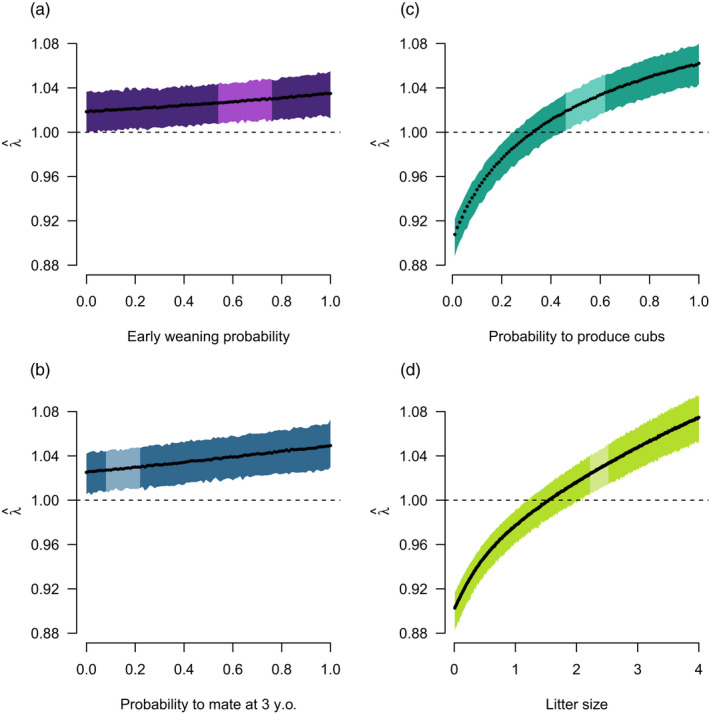
Response of the equilibrium growth rate ($\hat{\lambda }$) of the brown bear population in south‐central Sweden to changes in (a) the probability to wean cubs at the yearling stage (i.e., early weaning probability), (b) the probability for females aged three years to mate at this age and emerge from their winter den with cubs the following spring (i.e., probability to mate at three years old), (c) the probability for adult solitary females in the winter to emerge from their winter den with cubs the following spring (i.e., probability to produce cubs), and (d) litter size produced at each reproduction event. The lighter area in each panel represents the 95% confidence interval of the observed values for each reproductive trait (Table 1). Predictions are made assuming an annual hunting quota (q) of 5.5% and under the current hunting regulation of protecting family groups

With increasing simulated values of the four female reproductive traits investigated, $\hat{\lambda }$ increased (Figure 3). Population growth rate increased linearly with early weaning probability (Figure 3a) and with the probability to mate at three years of age (Figure 3b), but nonlinearly with the probability of adult females to produce cubs (Figure 3c) and with litter size (Figure 3d). The population increased ($\hat{\lambda }$ > 1) regardless of early weaning probability and probability to mate at three years old. However, the population was predicted to increase only when probability to produce cubs as adult was ≥0.33 [0.25, 0.45] and when litter size was ≥1.54 [1.19, 2.05], assuming q = 5.5% and under a regulation where family groups were protected from hunting.

### 3.4 Effects of hunting regulation

**FIGURE 4:**
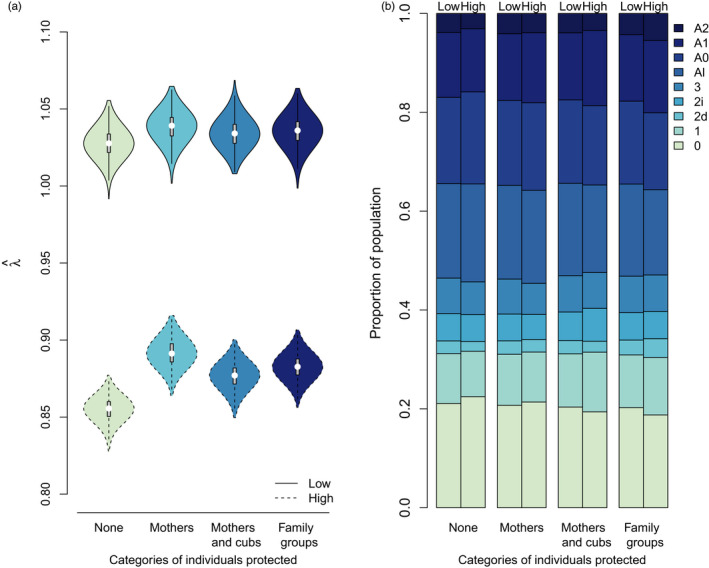
Effect of different hunting regulations based on which categories of individuals are afforded legal protection from hunting on (a) equilibrium growth rate ($\hat{\lambda }$) and (b) population stable stage structure at equilibrium at low (5%) *vs* high (20%) hunting quotas for brown bears in south‐central Sweden. The four hunting regulations are: (1) no individual is protected (“None”), (2) mothers are protected (“Mothers”), (3) mothers and their cubs are protected (“Mothers and cubs”), and (4) mothers and their offspring of any age are protected (“Family groups”). Definitions of female stages: 0 = female cubs, 1 = female yearlings, 2*i* = two‐year‐old females independent of their mother, 2*d* = two‐year‐old females dependent of their mother, 3 = three‐year‐old females, *Al* =adult solitary females, *A*0 = adult females with cubs, *A*1 = adult females with yearlings, and *A*2 = adult females with two‐year‐olds

Hunting regulation affected both the population structure $\hat{p}$ and growth rate $\hat{\lambda }$ (Figure 4). At low (5%) hunting quotas, hunting regulation affected the mean and distribution of $\hat{\lambda }$ only moderately, but when hunting quotas were high (20%), protecting certain categories of females within the population had a positive effect on$\hat{\lambda }$. However, $\hat{\lambda }$ did not increase linearly with the number of categories afforded protection. Indeed, the highest $\hat{\lambda }$ values were attained when only mothers, and not their offspring, were afforded legal protection (Figure 4a). Moreover, the effect of hunting regulation on the population stage structure was more apparent under high hunting quotas (Figure 4b). As protection from hunting was extended to more categories of individuals, there was an increase in the proportion of females forming extended family groups (stages 2*d*, *A*1, and *A*2). The effect was more pronounced under high hunting quotas and with the legal protection of family groups where the proportion of *A*2 and *Al* (solitary adult females) were the greatest and lowest, respectively.

### 3.5 Interplay between quotas, reproduction, and regulation

**FIGURE 5:**
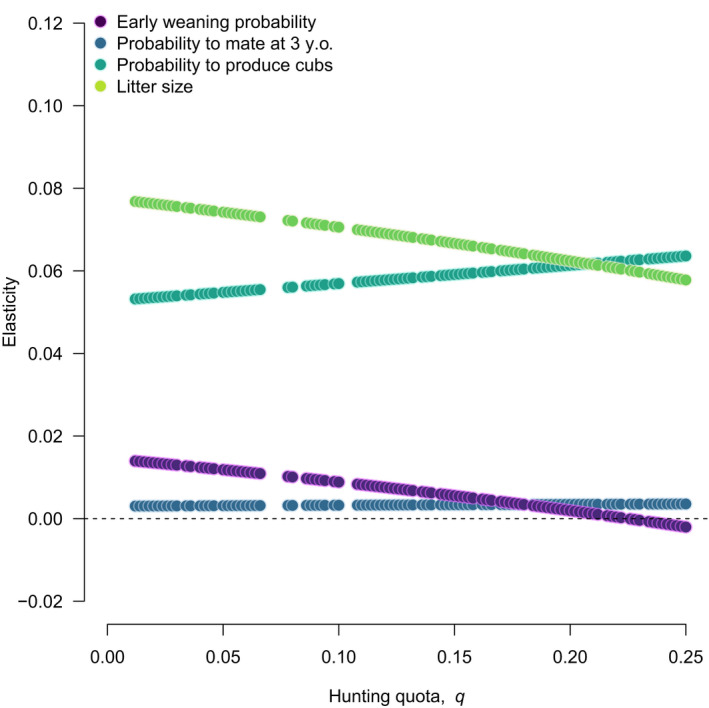
Elasticities of the equilibrium population growth rate to four brown bear female reproductive parameters under simulated values of hunting quotas, q. Note that here a q value of 0.05 corresponds to a 5% quota. Values above and below the dotted line indicate that an increase in the magnitude of the parameter would have a relative positive or a negative effect on population growth rate, respectively. Large values translate into a greater magnitude of the effect of a change in the reproductive rate on the population growth rate. Missing dots correspond to cases of convergence failure due to matrix singularity at simulated values

The elasticities of $\hat{\lambda }$ to reproductive traits changed with increasing hunting quotas (Figure 5). With increasing hunting quotas, the elasticity of population growth rate decreased for litter size and early weaning probability, but it increased for the probability to mate at three years old and the probability of producing cubs for adult females. Interestingly, the elasticity of population growth rate to early weaning probability became negative at very high hunting quotas. This suggests that whereas an increase in early weaning probability would increase population growth rate under low hunting quotas, a similar increase would reduce population growth rate at high hunting quotas.

**FIGURE 6:**
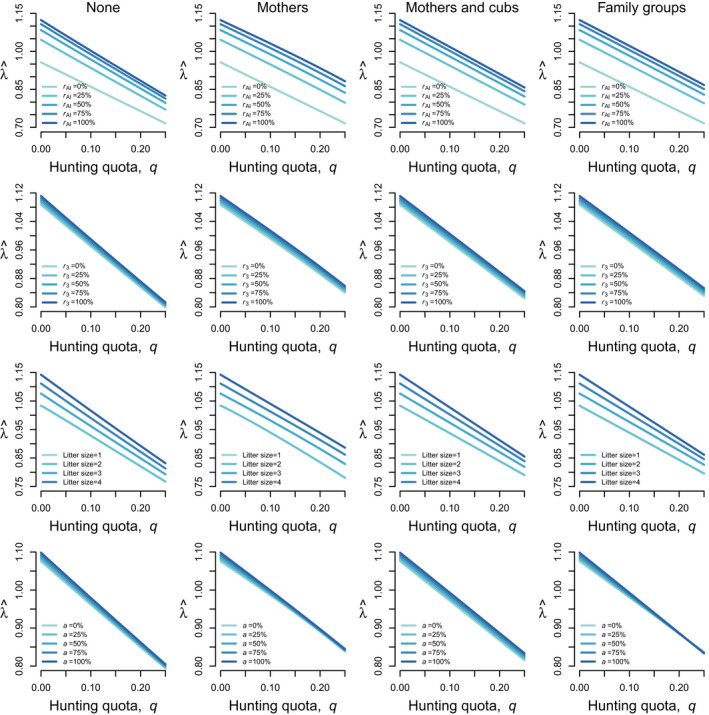
Interactive effects of hunting quotas, q, and changes in female brown bear reproductive traits on equilibrium population growth rate ($\hat{\lambda }$) under four scenarios of hunting regulation: (1) no individual is protected (column 1; “None”), (2) mothers are protected (column 2; “Mothers”), (3) mothers and their cubs are protected (column 3; “Mothers and cubs”), and (4) mothers and their offspring of any age are protected (column 4; “Family groups”). Hunting quotas were simulated over the range 0–0.25 (note that here a q value of 0.05 corresponds to a 5% quota), changes in the probability to produce cubs as adult ($r_{\mathrm{Al}}$; first row) probability to mate at three years old ($r_{3}$; second row), and early weaning probability a; fourth row) were simulated over the range 0–100%, and litter size (third row) was simulated over the range 1–4

Hunting quotas, reproductive trait values, and hunting regulations interacted in shaping equilibrium population growth rate, $\hat{\lambda }$ (Figure 6). Higher probabilities to produce cubs as adult ($r_{\mathrm{Al}}$), to mate at three years old ($r_{3}$), and larger litter sizes would all increase population growth rate at any given hunting quota. When no categories of individuals were protected, $\hat{\lambda }$declined sharply and tended to converge to very low values, regardless of changes in reproductive rates. However, when mothers and family groups were protected, increases in $r_{3}$, $r_{\mathrm{Al}}$, and litter size led to a slower decline in$\hat{\lambda }$. This effect was also detected under the legal protection of mothers and cubs, but only for changes in $r_{\mathrm{Al}}$ and $r_{3}$. Across hunting regulations, a high early weaning probability (a) was associated with greater $\hat{\lambda }$ at low hunting quotas. However, when hunting quotas increased, a higher early weaning probability led to similar $\hat{\lambda }$ when only mothers were protected and even greater $\hat{\lambda }$ at high hunting quotas when family groups were protected. See Appendix S1 (Fig. S1) for interaction plots using the full range of reproductive trait values.

## 4 DISCUSSION

Through the removal of individuals, human harvest directly impacts population size and, potentially, the persistence of wild populations (Jackson et al., 2001). Through selection on phenotypic traits, harvest can also indirectly impact population structure and dynamics (Frank et al., 2017; Law, 2000; Milner et al., 2007). Here, we quantified and compared the impact of hunting intensity, hunting regulation, and expected hunting‐induced selection on female reproductive traits on the population dynamics of brown bears. We found that the population should grow if hunting quotas are below 10% of the total population size annually under the current regulation protecting family groups. Among the reproductive traits considered, harvest‐induced selection acting on litter size and the probability for adult female brown bears to produce cubs would have the greatest impact on the population growth rate. Considering that sensitivity of population growth rate can be interpreted as a selection gradient (Caswell, 2008), our results show increasing selectivity for producing a litter (conditional on having the possibility to do so) with increasing hunting quotas. We also found that hunting regulations aimed at protecting the female segment of the population are effective, but providing legal protection to dependent offspring may dampen this effect. When family groups are protected, females producing litters are shielded against hunting, but this increases pressure on the other demographic groups that remain available for legal hunting.

Our model predicted an increase in the Swedish brown bear population, with an average 2.9% annual growth rate, which is similar to previous studies on the same population (Gosselin et al., 2015; Van de Walle et al., 2018), but relatively high compared with brown bear populations in North America (Garshelis et al., 2005). In Sweden, the current management objective is to reduce the size of the brown bear population (Swenson et al., 2017). Our results seem to suggest that this management objective was not met. However, hunting quotas used in our model (average of 5.5%) were estimated based on data from 1985 to 2013 (Swenson et al., 2017), and considering that hunting quotas and management kills have increased dramatically in recent years (Swenson et al., 2017), this is probably an underestimation. Therefore, our predicted 2.9% annual growth should be interpreted with caution, as it may represent an overestimation. In fact, using our last estimate of hunting quota of 11% in 2013 (Figure 2a), our model predicts an average population decline of about 3% annually (Figure 2c). This prediction is in line with recent estimates showing a decreasing trend in the Swedish brown bear population starting in 2008 (Swenson et al., 2017).

Because the levels of legal protection afforded to females and family groups can change over time (e.g., Swenson et al., 2017), a comprehensive comparison of the demographic impact of different hunting regulations under various hunting intensities is important for both the management of hunted populations and our understanding of their evolution. We found that hunting regulations aimed at protecting the female segment of the population are effective; by protecting mothers, the predicted population growth rate increases. However, this positive effect is mitigated when legal protection is extended to dependent offspring. Protecting dependent offspring produces a feedback by increasing hunting pressure on the fewer remaining categories of available individuals, which reduces population growth. This feedback effect would not have been captured using a deterministic, linear, population model (see Appendix S1). This result is in line with growing evidence that harvest affects populations through feedback loops (e.g., eco‐evo feedbacks; Govaert et al., 2019; Kokko & López‐Sepulcre, 2007) and calls for the consideration of frequency dependence when managing wild animal populations.

Perhaps the closest form of association among mammals is that between a mother and her dependent young. Their mutual influence on each others’ vital rates is apparent; a mother will typically have a positive effect on the survival of her dependent young (Clutton‐Brock, 1991; Klug et al., 2012), whereas dependent young may have a negative influence on their mother's fecundity, because in many species females do not enter estrus until their young are weaned (Borries et al., 2014). Additionally, young may also affect their mother's survival (if being part of a family unit can make her more or less vulnerable) and, in the case of extended associations, a mother may influence the fecundity of her offspring (e.g., reproductive suppression; Abbott, 1987). Demographic models should thus account for their intertwined fates (e.g., Hunter et al., 2010). In exploited populations, the association between a mother and her young can be even more important, as hunting regulations often afford protection for mothers, dependent young, or both, and can even reduce the cost of reproduction (e.g., Ericsson, 2001; Krofel et al., 2012; Solberg et al., 2000; Van de Walle et al., 2018). Such regulations can be motivated by population‐dynamic considerations, or to limit the effect of hunting on wild animal populations, as well as ethical concerns. In some cases, hunters avoid killing lactating female even though hunting regulation allows it (Rughetti & Festa‐Bianchet, 2014). There are various reasons for this, including concerns over potential negative effects for the population (many hunters keep the management of game populations in mind when hunting), sportsmanship (i.e., fair chase), and other ethical concerns that may have to do with the projection of anthropomorphic ideals (taboos, chivalry) onto wild populations. Despite the widespread potential for females and their dependent offspring to benefit from a legal, or ethical protection, seldom are the demographic consequences of those management actions quantitatively assessed. This may be due to the lack of comprehensive population models and the detailed data required to parameterize them. By incorporating cause‐ and season‐specific mortality rates, as well as interdependencies of mother–offspring vital rates, we were able to efficiently predict the outcome of various management decisions (e.g., increase or decrease in hunting quotas and changes in the regulations). Indeed, our results show the effectiveness of legal protection of reproductive females, especially at high hunting intensities, but also the potential for hunting‐induced selection on reproductive traits to dampen the effects of an increase in hunting quotas when mothers are protected. Extending the legal protection to all members of family groups has the additional potential to reverse selection gradient on age at weaning.

In ungulates, hunting rate is the most important factor driving the dynamics of exploited populations (Mysterud, 2011; Rughetti & Festa‐Bianchet, 2014; Rughetti et al., 2017). That is because, in long‐lived species, survival is typically the demographic rate with the highest elasticity and thus with the greatest potential to affect population growth (Gaillard et al., 1998). Here, we show that hunting rate in a large carnivore is also a key driver of population dynamics. Relative to other causes of mortality, population growth rate showed the greatest elasticity for hunting quotas, indicating that hunting has the largest impact on the dynamics of this population, compared with drivers of natural mortality. Our results also suggest that despite the protective effect of female reproductive traits, hunting‐induced selection for higher productivity in the population may not suffice to avoid population decline, even under strictly enforced legal protection of family groups. Reaching management goals for long‐lived species, such as the brown bear, is thus primarily dependent upon decisions on the level of hunting quotas to be issued, especially considering feedbacks from frequency‐dependent hunting mortality.

High levels of extrinsic mortality are expected to induce selection for increased productivity as a compensatory mechanism (Darimont et al., 2009; Law, 2000; Stearns, 1992). In support of this hypothesis, we found that brown bear females producing larger litters at an earlier age and more frequently would lead to increases in population growth rate. Increased productivity through high cub production relative to female size has likely contributed to the persistence of European brown bear populations after centuries of persecution (Zedrosser et al., 2011). Nevertheless, the importance of female reproductive tactics on population growth rate switched at low versus high hunting quotas: Weaning offspring at 1.5 years old would cause the population to increase at low hunting quotas, but to decrease at very high hunting quotas. Maintaining a family group over an extended period (2.5 years) shields females and their offspring against hunting mortality under the legal protection of family groups (which is the regulation currently in place in Sweden). This behavior is under selection at high hunting pressure, despite the reduced productivity associated with this reproductive tactic (Van dee Walle et al., 2018). Although we still detect a potential selection for longer periods of association between mother and offspring at high hunting intensity in this study, this effect was weaker due to the feedback effect of frequency dependence of hunting mortality. This is contrary to our expectation that frequency‐dependent mortality would strengthen the positive effect of longer maternal care at high hunting intensities. In fact, as individuals maintain family groups for longer periods, more individuals are unavailable for hunting, which results in an increased hunting intensity on the remaining categories of individuals. Nevertheless, later weaning would still shield females against hunting and offers a potential to reduce the negative impact of high hunting quotas.

We found that hunting quotas have a greater influence on hunted animal populations compared with hunting regulations in our study population. This is also the case for alpine chamois (*Rupicapra rupicapra*), for which hunting rates affected the population dynamics more strongly than hunting selectivity for nonlactating females (Rughetti & Festa‐Bianchet, 2014). The demographic consequences of hunting selectivity in the alpine chamois were only apparent under high simulated levels of hunting and selectivity. However, hunting regulations had measurable (albeit slight) demographic consequences even at low hunting quotas in our brown bear population, probably because the protection of lactating females in our system is strictly enforced and only a handful of mistakenly shot females with cubs have been reported over the last two decades in Sweden (Van de Walle et al., 2018). Our model also revealed that hunting selectivity on female reproductive traits has the potential to dampen the effect of harvest rates, which is in line with recent findings that selection can mitigate the effects of environmental changes on wild animal populations (Urban et al., 2020).

Like all modeling approaches, our predictions are based on a set of assumptions to reduce complexity, or because of the difficulty to estimate certain processes (Caswell, 2001). First, we did not account for potential age differences in vital rates within our adult female stages and used instead parameter estimates averaged for females aged between 6 and 10 years old (Bischof et al., 2018). Young adult females may still divert energy to growth and consequently show reduced reproductive output and survival probabilities due to life‐history trade‐offs (Stearns, 1992). Similarly, older females, through senescence, may show lower reproduction and survival (Kirkwood & Rose, 1991), however, considering that the onset of senescence in brown bears is ~27 years old (Schwartz et al., 2003) and that the average age at death is 4.8 years in Sweden (Bischof et al., 2008), this assumption appears reasonable. Second, we assumed that orphaned cubs would die. The assumption of death of orphaned offspring strongly affects the predicted demographic response of alpine chamois to selective hunting (Rughetti & Festa‐Bianchet, 2014). Because we did not capture and equip cubs with radio collars in our study, the fate of orphaned cubs is typically unknown. Some orphaned cubs have been documented to survive (Swenson et al., 1998), but considering that orphaned offspring can show reduced growth and future survival prospects (Festa‐Bianchet et al., 1994), their contribution to the population as adults might be limited (Zedrosser et al., 2013). Third, we assumed males and females as similarly vulnerable to hunting in our calculations of hunting mortality. This assumption is sensible in our study population considering that male and female brown bears are not discernable at a distance for hunters (Bischof et al., 2009), but can be challenged in other populations. Our results would potentially be reinforced in a male‐biased harvest system. Lastly, we assumed density independence as a previous study on the same population did not find relationships between bear density and demographic rates (Bischof et al., 2018). This may mean that the population has not reached stationarity, a phase regulated by density dependence where large populations decrease and small populations increase as resources (food, minerals, space, etc.) availability fluctuates (Coulson, 2020; Coulson et al., 2008). Nonstationarity is a reasonable assumption in the case of heavily hunted populations, but it may not hold for all populations, especially if removal rates are low. Despite those assumptions, our model predicts population growth rates within the range of published estimates for this population of brown bears and others (Garshelis et al., 2005; Gosselin et al., 2015; Kindberg et al., 2011; Van de Walle et al., 2018). As such, it represents a valuable tool for the management of this and other brown bear populations.

As the human impact on wild species is increasing, it is critical to predict the consequence of those changes on population dynamics and evaluate the effects of novel selective pressures on the fate of populations (Lasky et al., 2020). Models that include both human‐driven extrinsic mortality, as well as phenotypic trait changes, are therefore useful to explore the effect of multi‐trait life‐history changes in natural populations. In fisheries, harvest‐induced evolution is expected to increase the population growth rate in such a way that populations adapted to harvest would support higher levels of harvest (Dunlop et al., 2015; Enberg et al., 2009; Heino et al., 2013). In hunted populations of long‐lived species, there is less evidence of harvest‐induced evolutionary changes (Pigeon et al., 2016), potentially because they may require a longer period to detect. A critical step would therefore be to expand population models to integrate and quantify the possible feedback of evolutionary changes in life‐history traits on population processes (Govaert et al., 2019; Smallegange & Coulson, 2013) especially since evolution can offer a mean to promote population resilience to exploitation (Dunlop et al., 2015). Notwithstanding, what matters the most in terms of demography is to understand the consequences of phenotypic changes, regardless of their cause (Hendry, 2016). As such, phenotypic changes ought to be considered in management and conservation of wild populations, especially as they may offer mechanisms for mitigating the negative consequences of intense harvest.

## CONFLICT OF INTEREST

None declared

## Supporting information

Appendix S1

## Data Availability

The MATLAB codes used for the model construction and analyses are available in the Appendix S1 of this article. The data that support the findings of this study are available upon request in figshare at https://usn.figshare.com/articles/dataset/Van_de_Walle_et_al_2021_Evolutionary_Applications/14459376.
