# Supplementary material for: The interplay between hunting rate, hunting selectivity, and reproductive strategies shapes population dynamics of a large carnivore
Source: Evol Appl. 2021 Jun 2;14(10):2414–32. doi: 10.1111/eva.13253 (PMC8549626; doi:10.1111/eva.13253)
Supplement: Supplementary file 1 — Appendix S1 [file EVA-14-2414-s001.docx]

**The interplay between hunting rate, hunting selectivity, and reproductive strategies shapes population dynamics of a large carnivore**

Supplementary materials

## Supplementary Material 1: Supplementary tables and figures

**Table S1:** Description and formulation of transition probabilities, *G_i,j,k_*, between female stages (from stage *i* to stage *j*) for each season (*k*). Definitions of female stages: 0 = female cubs, 1 = female yearlings, 2i = two-year-old females independent of their mother, 2d = two-year-old females dependent of their mother, 3 = three-year-old females, Al = adult solitary females, A0 = adult females with cubs, A1 = adult females with yearlings, and A2 = adult females with two-year-olds. Definitions of seasons: s = spring, f = fall, w = winter. Definitions of other terms: *S_i,k_* = survival rate of individuals in stage *i* at the beginning of the season *k*, *a* = early weaning probability, *l_k_* = probability to lose an entire litter of cubs during season *k*, *y_k_* = probability to lose an entire litter of yearlings during season *k*, *r_3_* = Probability to mate as a three-year-old and emerge from the den with cubs the following spring, *r_Al_* = probability that a solitary adult female emerges from the den with cubs the following spring, *ncub_s_* = number of cubs produced in the spring.

| Transition | Description | Equation |
| --- | --- | --- |
| $G_{0,0,s}$ | Probability that a cub in the spring remains a cub in the fall. | $G_{0,0,s}= S_{A0,s}\times S_{0,s}$ |
| $G_{1d,1d,s}$ | Probability that a (dependent) yearling in the spring remains a dependent yearling in the fall. | $G_{1d,1d,s}= (1-a)\times S_{A1,s}\times S_{1d,s}$ |
| $G_{1d,1i,s}$ | Probability that a (dependent) yearling in the spring becomes an independent yearling in the fall. | $G_{1d,1i,s}= a\times S_{1i,s}$ |
| $G_{2d,2i,s}$ | Probability that a dependent two-year-old in the spring becomes an independent two-year-old in the fall. | $G_{2d,2i,s}= S_{2,s}$ |
| $G_{2i,2i,s}$ | Probability that an independent two-year-old in the spring remains an independent two-year-old in the fall. | $G_{2i,2i,s}= S_{2,s}$ |
| $G_{3,3,s}$ | Probability that a three-year-old in the spring remains a three-year-old in the fall. | $G_{3,3,s}= S_{3,s}$ |
| $G_{Al,Al,s}$ | Probability that a solitary female in the spring remains a solitary female in the fall. | $G_{Al,Al,s}= S_{Al,s}$ |
| $G_{A0,Al,s}$ | Probability that a female with cubs in the spring loses her litter and becomes a solitary female in the fall. | $G_{A0,Al,s}= {l_{s}\times S}_{Al,s}$ |
| $G_{A0,A0,s}$ | Probability that a female with cubs in the spring keeps her litter and remains a female with cubs in the fall. | $G_{A0,A0,s}= {{(1-l}_{s})\times S}_{A0,s}$ |
| $G_{A1,Al,s}$ | Probability that a female with yearlings in the spring weans her litter and becomes a solitary female in the fall. | $G_{Al,Al,s}= {a\times S}_{Al,s}$ |
| $G_{A1,A1,s}$ | Probability that a female with yearlings in the spring keeps her litter and remains a female with yearlings in the fall. | $G_{A1,A1,s}= {(1-a)\times S}_{A1,s}$ |
| $G_{A2,Al,s}$ | Probability that a female with two-year-olds in the spring weans her litter (at this stage this probability is 1) and becomes a solitary female in the fall. | $G_{A2,Al,s}= S_{Al,s}$ |
| $G_{0,0,f}$ | Probability that a female cub in the fall remains a cub in the winter. | $G_{0,0,f}=S_{0,f}\times S_{A0,f}$ |
| $G_{1d,1d,f}$ | Probability that a dependent yearling female in the fall remains a dependent yearling female in the winter. | $G_{1d,1d,f}=S_{A1,f}\times S_{1d,f}$ |
| $G_{1d,1i,f}$ | Probability that a dependent yearling in the fall is weaned and becomes independent in the winter. | $G_{1d,1i,f}=(1-S_{A1,f})\times S_{1i,f}$ |
| $G_{1i,1i,f}$ | Probability that an independent yearling in the fall remains a dependent yearling in the winter. | $G_{1i,1i,f}=S_{1i,f}$ |
| $G_{2i,2i,f}$ | Probability that an independent two-year-old female in the fall remains an independent two-year-old in the winter. | $G_{2i,2i,f}=S_{2,f}$ |
| $G_{3,3,f}$ | Probability that a three-year-old female in the fall remains a three-year-old female in the winter. | $G_{3,3,f}=S_{3,f}$ |
| $G_{Al,Al,f}$ | Probability that a solitary female in the fall remains a solitary female in the winter. | $G_{Al,Al,f}=S_{Al,f}$ |
| $G_{A0,Al,f}$ | Probability that a female with cubs in the fall loses her litter and become a solitary female in the winter. | $G_{A0,Al,f}=l_{f}\times S_{Al,f}$ |
| $G_{A0,A0,f}$ | Probability that a female with cubs in the fall keeps her litter and remains a female with cubs in the winter. | $G_{A0,A0,f}=(1-l_{f})\times S_{A0,f}$ |
| $G_{A1,Al,f}$ | Probability that a female with yearlings in the fall loses her litter and becomes a solitary female in the winter. | $G_{A1,Al,f}=y_{f}\times S_{Al,f}$ |
| $G_{A1,A1,f}$ | Probability that a female with yearlings in the fall keeps her litter and remains a female with yearlings in the winter. | $G_{A1,A1,f}=(1-y_{f})\times S_{A1,f}$ |
| $G_{0,1,w}$ | Probability that a female cub in the winter becomes a dependent yearling cub the following spring | $G_{0,1,w}=S_{A0,w}\times S_{0,w}$ |
| $G_{1d,2d,w}$ | Probability that a dependent yearling female in the winter becomes a dependent two-year-old female in the following spring. | $G_{1d,2d,w}=S_{A1,w}\times S_{1d,w}$ |
| $G_{1d,2i,w}$ | Probability that a dependent yearling female in the winter becomes an independent two-year-old female in the following spring. | $G_{1d,2i,w}=(1-S_{A1,w})\times S_{1i,w}$ |
| $G_{1i,2i,w}$ | Probability that an independent yearling female in the winter becomes an independent two-year-old female in the following spring. | $G_{1i.2i,w}=S_{1i,w}$ |
| $G_{2i,3,w}$ | Probability that an independent two-year-old female in the winter becomes a three-year-old female in the following spring. | $G_{2i,3,w}=S_{2,w}$ |
| $G_{3,Al,w}$ | Probability that a three-year-old female in the winter becomes a solitary adult female in the following spring. | $G_{3,Al,w}=S_{3,w}\times(1-r_{3})$ |
| $G_{3,A0,w}$ | Probability that a three-year-old female in the winter becomes an adult female with cubs in the following spring. | $G_{3,A0,w}=S_{3,w}\times r_{3}$ |
| $G_{Al,Al,w}$ | Probability that a solitary adult female in the winter remains a solitary adult female in the following spring. | $G_{Al,Al,w}=S_{Al,w}\times(1-r_{Al})$ |
| $G_{Al,A0,w}$ | Probability that a solitary adult female in the winter becomes a female with cubs the following spring. | $G_{Al,A0,w}=S_{Al,w}\times r_{Al}$ |
| $G_{A0,Al,w}$ | Probability that a female with cubs in the winter loses her litter and becomes a solitary female the following spring. | $G_{A0,Al,w}=l_{w}{\times S}_{Al,w}$ |
| $G_{A0,A1,w}$ | Probability that a female with cubs in the winter keeps her cubs and become with yearlings the following spring. | $G_{A0,A1,w}=(1-l_{w}){\times S}_{A0,w}$ |
| $G_{A1,Al,w}$ | Probability that a female with yearlings in the winter loses her litter and becomes a solitary adult female the following spring. | $G_{A1,Al,w}=y_{w}\times S_{Al,w}$ |
| $G_{A1,A2,w}$ | Probability that a female with yearlings keeps her litter and become a female with two-year-olds the following spring. | $G_{A1,A2,w}=(1-y_{w})\times S_{A1,w}$ |
| $f_{3}$ | Fertility of three-year-old females | $f_{3}=r_{3}\times{ncub}_{s}\times\frac{1}{2}$ |
| $f_{Al}$ | Fertility of solitary adult females | $f_{Al}=r_{Al}\times{ncub}_{s}\times\frac{1}{2}$ |

**Table S2:** Derived parameters used in the transitions from Table S1. Definitions of female stages: 0 = female cubs, 1 = female yearlings, 2i = two-year-old females independent of their mother, 2d = two-year-old females dependent of their mother, 3 = three-year-old females, Al = adult solitary females, A0 = adult females with cubs, A1 = adult females with yearlings, and A2 = adult females with two-year-olds. Definitions of seasons: s = spring, f = fall, w = winter. Definitions of other terms: *S_i,k_* = survival rate of individuals in stage *i* at the beginning of the season *k*, *a* = early weaning probability, *l_k_* = probability to lose an entire litter of cubs during season *k*, *y_k_* = probability to lose an entire litter of yearlings during season *k*, *r_3_* = probability that a three-year-old female emerges from the den with cubs, *r_Al_* = probability that a solitary adult female emerges from the den with cubs, *ncub_k_* = number of cubs at season *k*, *nyearl_k_ =* number of yearlings at season *k*. Note that parameters relating to stage-specific hunting mortality rates, *h_i_*, were either set to 0 or *h*, depending on the scenario considered (Table 3) and following equation 2 in the main text.

| Parameter | Description | Equation |
| --- | --- | --- |
| ${ncub}_{f}$ | Number of cubs within a litter in the fall. | ${ncub}_{f}={ncub}_{s}\times S_{0,s}$ |
| ${ncub}_{w}$ | Number of cubs within a litter in the winter. | ${ncub}_{w}={ncub}_{f}\times S_{0,f}$ |
| $l_{s}$ | Probability that a female loses her entire litter of cubs during the spring. | $l_{s}=\left( 1-S_{0,s} \right)^{{ncub}_{s}}$ |
| $l_{f}$ | Probability that a female loses her entire litter of cubs during the fall. | $l_{f}=\left( 1-S_{0,f} \right)^{{ncub}_{f}}$ |
| $l_{w}$ | Probability that a female loses her entire litter of cubs during the winter. | $l_{w}=\left( 1-S_{0,w} \right)^{{ncub}_{w}}$ |
| ${nyearl}_{s}$ | Number of yearlings within a litter in the spring. | $nyearl_{s}={ncub}_{w}\times S_{0,w}$ |
| ${nyearl}_{f}$ | Number of yearlings within a litter in the fall. | $nyearl_{f}=nyearl_{s}\times S_{1d,s}$ |
| ${nyearl}_{w}$ | Number of yearlings within a litter in the winter. | $nyearl_{w}=nyearl_{f}\times S_{1d,f}$ |
| $y_{f}$ | Probability that a female loses her entire litter of yearlings during the fall. | $y_{f}=\left( 1-S_{1d,f} \right)^{nyearl_{f}}$ |
| $y_{w}$ | Probability that a female loses her entire litter of yearlings during the winter. | $y_{w}=\left( 1-S_{1d,w} \right)^{nyearl_{w}}$ |
| $S_{1d,s}$ | Survival probability of dependent yearling females during the spring. | $S_{1d,s}=(1-w_{1d,s})$ |
| $S_{1d,f}$ | Survival probability of dependent yearling females during the fall. | $S_{1d,f}=(1-w_{1d,f}-h_{1d})$ |
| $S_{1d,w}$ | Survival probability of dependent yearling females during the winter. | $S_{1d,w}=(1-w_{1d,w})$ |
| $S_{1i,s}$ | Survival probability of independent yearling females during the spring. | $S_{1i,s}=(1-w_{1i,s})$ |
| $S_{1i,f}$ | Survival probability of independent yearling females during the fall. | $S_{1i,f}=(1-w_{1i,f}-h_{1i}$) |
| $S_{1i,w}$ | Survival probability of independent yearling females during the winter. | $S_{1i,w}=(1-w_{1i,w})$ |
| $S_{2,s}$ | Survival probability of 2-year-old females during the spring. | $S_{2,s}=(1-w_{2,s})$ |
| $S_{2,f}$ | Survival probability of 2-year-old females during the fall. | $S_{2,f}=(1-w_{2,f}-h_{2}$) |
| $S_{2,w}$ | Survival probability of 2-year-old females during the winter. | $S_{2,w}=(1-w_{2,w})$ |
| $S_{3,s}$ | Survival probability of 3-year-old females during the spring. | $S_{3,s}=(1-w_{3,s})$ |
| $S_{3,f}$ | Survival probability of 3-year-old females during the fall. | $S_{3,f}=(1-w_{3,f}-h_{3})$ |
| $S_{3,w}$ | Survival probability of 3-year-old females during the winter. | $S_{3,w}=(1-w_{3,w})$ |
| $S_{Al,s}$ | Survival probability of solitary females during the spring. | $S_{Al,s}=\left( 1-w_{Al,s} \right)$ |
| $S_{Al,f}$ | Survival probability of solitary females during the fall. | $S_{Al,f}=(1-w_{Al,f}-h_{Al})$ |
| $S_{Al,w}$ | Survival probability of solitary females during the winter. | $S_{Al,w}=\left( 1-w_{Al,w} \right)$ |
| $S_{A0,s}$ | Survival probability of females with cubs during the spring. | $S_{A0,s}=(1- w_{A0,s})$ |
| $S_{A0,f}$ | Survival probability of females with cubs during the fall. | $S_{A0,f}=(1- w_{A0,f}-h_{A0})$ |
| $S_{A0,w}$ | Survival probability of females with cubs during the winter. | $S_{A0,w}=(1- w_{A0,w})$ |
| $S_{A1,s}$ | Survival probability of females with yearlings during the spring. | $S_{A1,s}=(1-w_{A1,s})$ |
| $S_{A1,f}$ | Survival probability of females with yearlings during the fall. | $S_{A1,f}=(1-w_{A1,f}-h_{A1})$ |
| $S_{A1,w}$ | Survival probability of females with yearlings during the winter. | $S_{A1,w}=(1-w_{A1,w})$ |

**Table S3**: Changes in stage-specific mortality rates (or survival rates for stage 0; cubs) due to hunting under the four scenarios of hunting regulation considered for the population model of brown bear in south-central Sweden: (1) no individual is protected, (2) mothers but not dependent offspring are protected, (3) mothers and dependent cubs are protected, and (4) entire family groups are protected (i.e., mothers and dependent offspring of any age).

| Parameter | Scenario 1  *None* | Scenario 2  *Females* | Scenario 3  *Females and cubs* | Scenario 4  *Family groups* |
| --- | --- | --- | --- | --- |
| $S_{0,f}$ | 1−ℎ | ℎ | Set to 1 | Set to 1 |
| $h_{1d}$ | ℎ | ℎ | ℎ | Set to 0 |
| $h_{1i}$ | ℎ | ℎ | ℎ | ℎ |
| $h_{2}$ | ℎ | ℎ | ℎ | ℎ |
| $h_{3}$ | ℎ | ℎ | ℎ | ℎ |
| $h_{Al}$ | ℎ | ℎ | ℎ | ℎ |
| $h_{A0}$ | ℎ | Set to 0 | Set to 0 | Set to 0 |
| $h_{A1}$ | ℎ | Set to 0 | ℎ | Set to 0 |


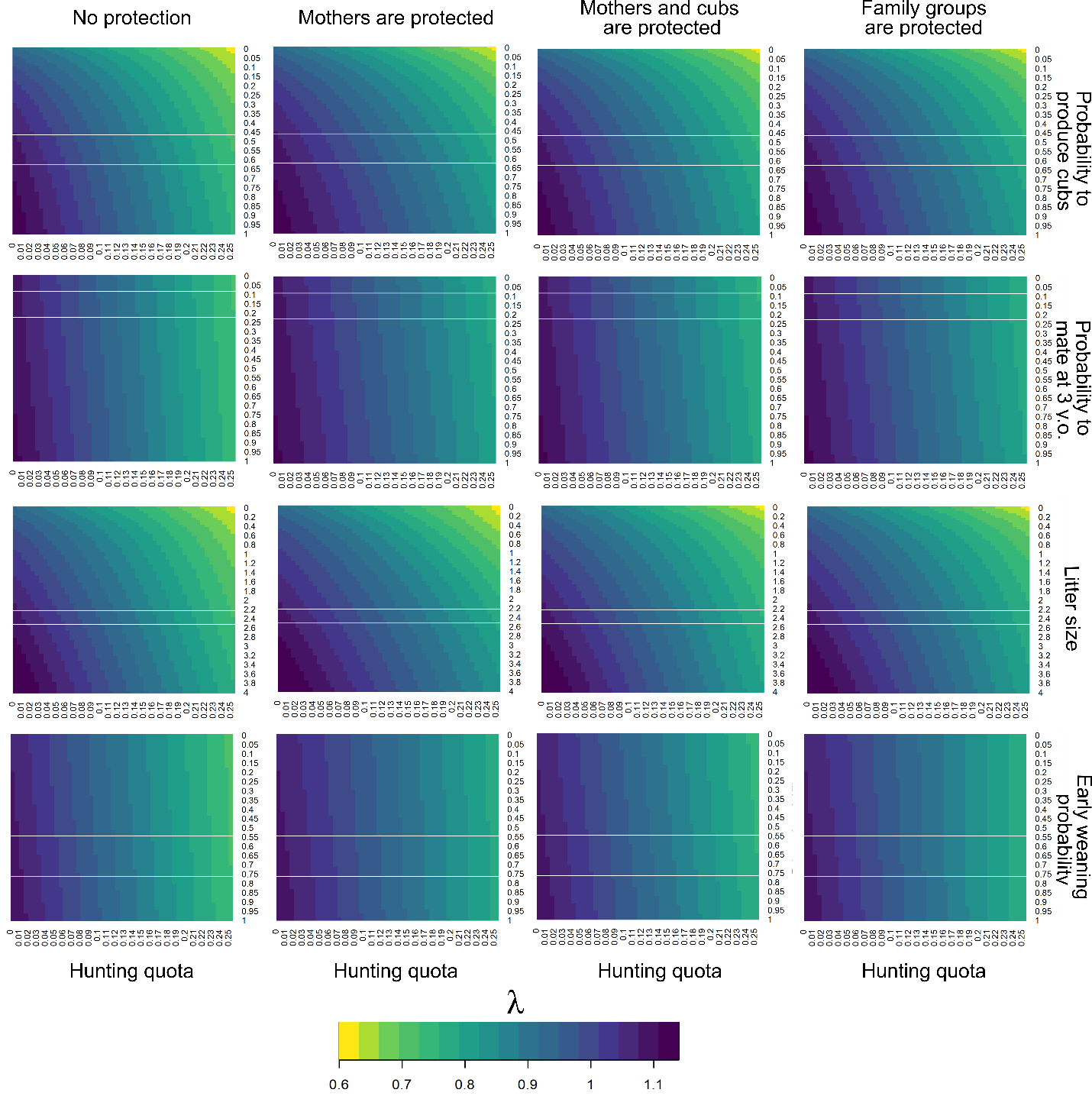


**Figure S1:** Interactive effects of hunting quota and changes in female brown bear reproductive

traits under four scenarios of hunting regulation: 1) no categories of bears are protected (“No protection”), 2) mothers but not offspring are protected, 3) mothers and cubs are protected, and 4) mothers and offspring of any age are protected. Each cell in the matrices indicates the

equilibrium population growth rate estimated for a given hunting quota and reproductive trait value. Within the white vertical lines are the 95 % confidence intervals of observed parameter estimates. Note that a *q* value of 0.05 correspond to a 5% quota.

Supplementary Material 2: Effect of frequency dependence feedback

Here we assessed the effect of omitting frequency-dependence of hunting mortality in the matrix population model of brown bears in south-central Sweden. To do so, we “turned off” the calculation of the parameter hunting mortality, *h*, and attributed a fixed hunting pressure on the available categories of individuals. In the frequency-dependent version of the model, *h* is calculated based on a hunting quota (proportion of total population size that can be harvested annually) and the ratio of unavailable to available bears in the population (see the main text). Because of the feedback loop (See Figure 1A in the main text), *h* varies over time. In the deterministic (no frequency-dependence) version of the model, we had to fix *h* for the available categories of individuals in a way that results would be comparable between the frequency-dependent and the deterministic population models. We extracted *h* values at equilibrium corresponding to two values of *q* used in Fig. 4 (main text): 5 % (low) 20 % (high). We used those corresponding *h* (low: 8 %; high: 27 %) in the deterministic version of the model and applied them to the available categories of individuals. In the absence of frequency dependence, the level of protection from hunting afforded to mothers and dependent offspring affected the population growth rate differently compared to when considering frequency-dependence. At a low hunting rate, the population switches from declining to increasing when mothers are afforded legal protection (Fig. S2A). Beyond this level of protection, population growth rate remains relatively unchanged. However, at a high hunting rate, there is a steep and relatively constant increase in asymptotic population growth rate with increasing level of protection on females and dependent offspring. This contrasts with the model including frequency dependence, where protecting mothers only, and not their dependent offspring, yields the highest population growth rate (see Figure 4A in the main text). However, the stable stage structure was similar in both model versions (Fig. S2B and Fig. 4B).


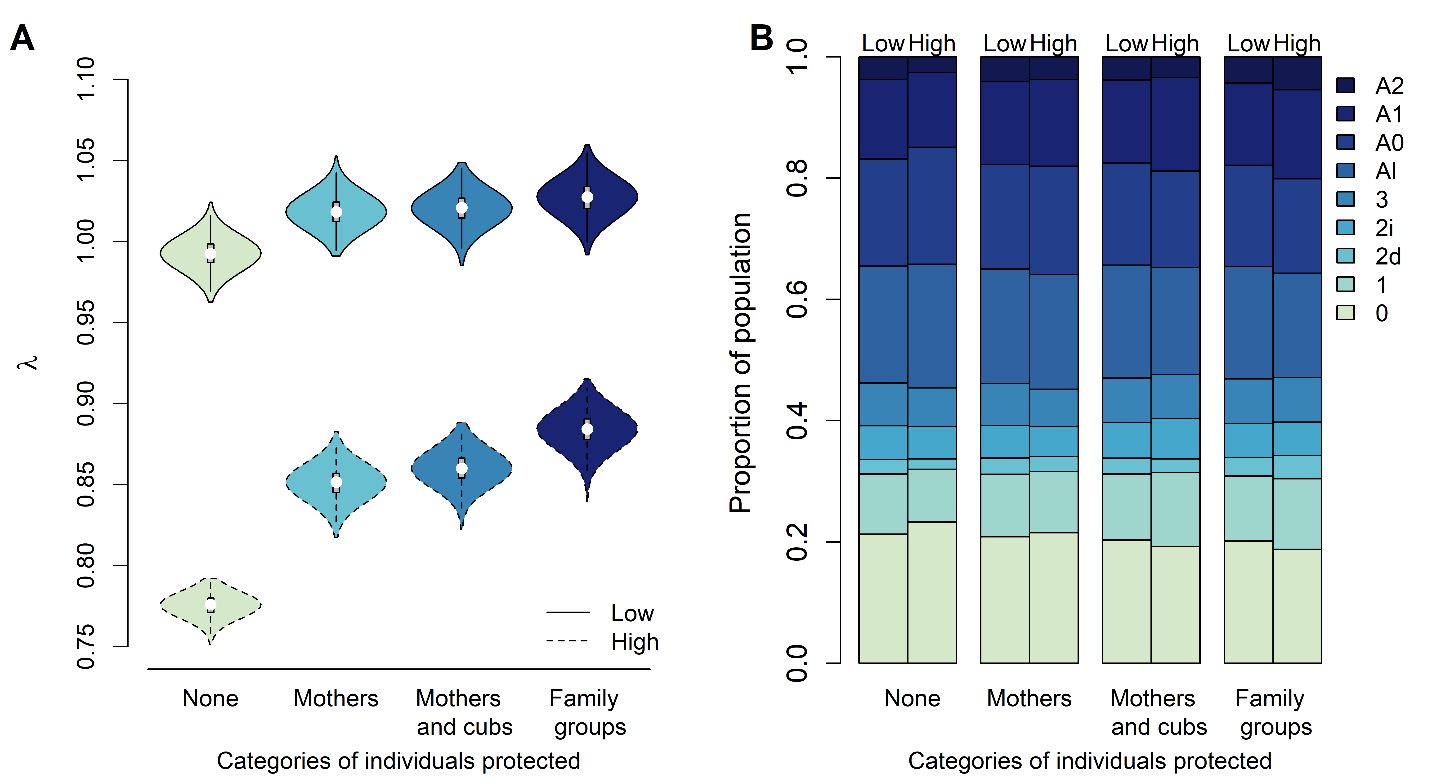


**Figure S2.** Effect of different hunting regulations based on which categories of individuals are afforded legal protection from hunting on A) population growth rate (λ) and B) population stable stage structure under low (8 %) *vs* high (27 %) hunting mortality rates for brown bears in south-central Sweden. Definitions of female stages: 0 = female cubs, 1 = female yearlings, 2i = two-year-old females independent of their mother, 2d = two-year-old females dependent of their mother, 3 = three-year-old females, Al = adult solitary females, A0 = adult females with cubs, A1 = adult females with yearlings, and A2 = adult females with two-year-olds.
